# Piloting a manualised weight management programme (Shape Up-LD) for overweight and obese persons with mild-moderate learning disabilities: study protocol for a pilot randomised controlled trial

**DOI:** 10.1186/1745-6215-14-71

**Published:** 2013-03-12

**Authors:** Rebecca J Beeken, Dimitrios Spanos, Sally Fovargue, Rachael Hunter, Rumana Omar, Angela Hassiotis, Michael King, Jane Wardle, Helen Croker

**Affiliations:** 1 Department of Epidemiology & Public Health, University College London, 1-19 Torrington Place, London, WC1E 6BT, UK; 2 Anglian Community Enterprise, Mill Road Therapy Centre, Colchester, CO45LJ, UK; 3 Department of Primary Care & Population Health, University College London, Gower Street, London, WC1E 6BT, UK; 4 Department of Statistical Science, University College London, Gower Street, London, WC1E 6BT, UK; 5 Department of Mental Health Sciences, University College London, Gower Street, London, WC1E 6BT, UK

**Keywords:** Learning disabilities, Weight management, Overweight

## Abstract

**Background:**

National obesity rates have dramatically risen over the last decade. Being obese significantly reduces life expectancy, increases the risk of a range of diseases, and compromises quality of life. Costs to both the National Health Service and society are high. An increased prevalence of obesity in people with learning disabilities has been demonstrated. The consequences of obesity are particularly relevant to people with learning disabilities who are already confronted by health and social inequalities. In order to provide healthcare for all, and ensure equality of treatment for people with learning disabilities, services must be developed specifically with this population in mind. The aim of this project is to pilot the evaluation of a manualised weight management programme for overweight and obese persons with mild-moderate learning disabilities (Shape Up-LD).

**Methods/Design:**

An individually randomised, controlled pilot trial in 60 overweight and obese (body mass index ≥ 25) adults (age ≥ 18) with mild-moderate learning disabilities and their carers will be carried out, comparing “Shape Up-LD” with usual care. The manualised Shape Up-LD intervention will involve 12 weekly sessions, which include healthy eating messages, advice on physical activity and use of behaviour change techniques to help people manage their weight. Assessments of participants will be conducted at baseline, 12 weeks and 6 months. Service users and their carers and service providers will also give their perspectives on the experience of Shape Up-LD in qualitative interviews at 12 weeks. Feasibility outcomes will include recruitment rates, loss to follow-up, compliance rates, completion rates, collection of information for a cost-effectiveness analysis and an estimation of the treatment effect on weight.

**Discussion:**

The findings from this study will inform our preparation for a definitive randomised controlled trial to test the efficacy of the programme with respect to weight loss and maintenance in this population. Weight loss through Shape Up-LD could lead to improvements in health and quality of life. Costs to the National Health Service might be reduced through decreased overall service use because of improved health. The programme would also ensure a more equitable service for overweight service users with learning disabilities and fill the current gap in weight management services for this population.

**Trial registration:**

International Standard Randomised Controlled Trial No ISRCTN39605930

## Background

Obesity rates have risen in the UK over the last decade [1]. Being obese reduces life expectancy and increases the risk of a range of conditions including cardiovascular disease and type 2 diabetes [2]. Obese individuals report decreased quality of life and face discrimination in healthcare, education and workplace settings [3]. The National Health Service (NHS) was estimated to spend £2.3 billion in 2007 on costs directly related to obesity, with wider costs to society reaching £15.8 billion [4].

An increased prevalence of obesity in people with learning disabilities has been demonstrated in a community study of 945 adults with learning disabilities, where 39% of women and 28% of men were obese compared with 25% of women and 23% of men in the general population [5]. Several factors are thought to contribute to the increased risk of overweight and obesity in people with learning disabilities, including physical inactivity and low adherence to healthy diets [6]. The consequences of obesity are particularly relevant to people with learning disabilities, who are already confronted by health and social inequalities [7]. Obesity can exacerbate disabling conditions and has been shown to contribute to the already reduced life expectancy of people with learning disabilities [7].

In order to provide healthcare for all and ensure equality of treatment for people with learning disabilities, services must be developed specifically with this population in mind, taking into account differences in health needs, communication problems and understanding [7]. For those individuals who rely on substantial care, carers’ attitudes and knowledge may also need to be addressed [8-10]. The National Institute for Health and Clinical Excellence (NICE) Obesity guidelines [11] recognise that the prevalence of obesity is higher in this group and that there are difficulties of access to information and support. It recommends the inclusion of behavioural management principles in lifestyle interventions to address the issue.

A recent review of weight management interventions for adults with learning disabilities [6] revealed a paucity of studies, identifying only eight, and there have since been a few more recent studies [12,13]. Two of the studies described in Hamilton [6] solely focused on increasing physical activity levels and had minimal impact on weight outcomes [14,15]. The six other studies drew on behavioural management principles, in line with the generic guidance for adult weight management. Four of these [16-19] were based in the US, and all are relatively old studies. Although they included only small numbers of participants, they broadly found positive effects on weight loss. The two remaining studies [20,21] were conducted in the UK. One consisted of nurse-led health promotion sessions [20], which did not specifically target obese individuals, but those who were overweight experienced a significant mean reduction in weight of 3.4 kg.

The final study [21] compared the impact of input from a ‘Healthy Living Coordinator’ with a ‘no input’ group. This was the only study to include a relatively large sample (*n* = 83). Few details are given regarding the nature of the intervention, but it appeared to be largely educational, with some guidance to support behaviour changes (e.g. planning, social support and motivation). There was a small reduction in weight (−1.5 kg) in the intervention group at 12 months and significant increase in weight for the “no input” group (0.9 kg). A longer term follow-up to this study [22] found reductions in body mass index (BMI) in the intervention group over 6 years, but these were not significantly different from changes in the control group. Another review [23] was in broad agreement with these findings and additionally included one qualitative study, which suggested such interventions are received positively by this population group.

The evidence outlined here supports the use of behavioural/educational interventions with strategies to support behaviour change for obese individuals with learning disabilities. However, most existing studies have limitations. The majority are on small samples and have no control group. Despite this, there are indications for potentially useful strategies to include in interventions, for example, weight monitoring, teaching self-control strategies, external reinforcement and guidance about planning.

The weight management programme we intend to trial was developed by dietitians, health psychologists and specialists in learning disabilities with input from service users, and it includes the strategies identified by previous research alongside education. Trialling this programme on a large sample with a control group will give a clear indication of the effectiveness of the programme and its potential to fill the current gap in weight management services for people with learning disabilities. In line with Phase 2 of the Medical Research Council (MRC) complex interventions framework [24], the aim of this study is to pilot such a trial.

### Study objectives

The key research question is:

Can we design a feasible, large-scale, randomised controlled trial (RCT) that will answer the following question: Is Shape Up-LD more effective than usual care in helping overweight and obese service users with mild-moderate learning disabilities reduce body weight?

In order to answer this question, we will conduct a small randomised controlled trial comparing the use of Shape Up-LD with usual care within two inner London learning disabilities services. The RCT will include a number of potential secondary outcome measures in addition to the primary outcome measure [reduction in weight (kg)].

This RCT will enable us to:

(1) measure the eligible numbers, recruitment rate, loss to follow-up, compliance and weight SD of participating overweight and obese service users within two inner London learning disabilities services.

(2) determine the acceptability of randomisation to service users through its effect on dropout rates.

(3) determine, through completion rates, the appropriateness and the acceptability of the outcome measures to service users in order to see whether, in addition to our primary outcome, we can also explore changes in body fat, waist circumference, blood pressure, mental health, quality of life, health and social care resource use, self-esteem, health knowledge and behaviours.

(4) measure compliance of the facilitators to the manualised intervention

(5) estimate the treatment effect with confidence intervals (CI) to determine whether the CI includes a clinically important effect, which will support the need to conduct the full trial.

We will also conduct qualitative interviews with service users, their carers and service providers to assess the acceptability of the treatment and to explore their experience of the treatment including any barriers and/or facilitators to adherence. Findings from these interviews also will enable us to refine the Shape Up-LD intervention further.

## Methods/Design

### Design

**Figure 1:**
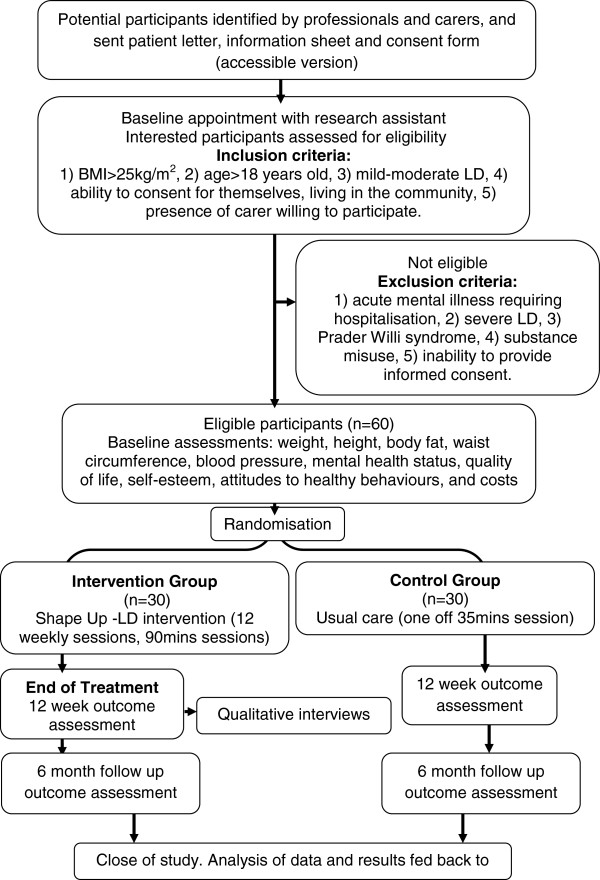
Flow chart.

The trial is a two-arm, individually randomised, controlled pilot trial in overweight and obese adults with mild-moderate learning disabilities comparing “Shape Up-LD” with usual care. Figure 1 illustrates the pathway through the trial. This trial corresponds to Phase 2 of the MRC’s guidelines for complex interventions: assessing feasibility [24].

### Recruitment

#### Participants

Adult service users with mild-moderate learning disabilities from two inner London community services.

#### Recruitment strategy

There are at least 450 service users with mild-moderate learning disabilities in our target areas. Our field testing of the programme with a small group of service users in North East Essex suggested that it is suitable to include service users who are both overweight and obese (as opposed to restricting the programme to only obese users). Therefore, based on the estimated prevalence rates of obesity and overweight (over 40%) in this population, at least 180 of these are likely to be eligible for the study. As BMI and weight generally go unrecorded in this population, potential participants will be self-referred or referred by their paid or informal carers or professionals in response to posters advertising the study placed in the reception of the services and in local day care facilities for people with learning disabilities.

Individuals who are interested will be checked for eligibility and those who fit the inclusion criteria will be invited to participate via a letter containing the information sheet. Recruitment will take place over a 12-month period, with service users or service user-primary paid/informal carer dyads recruited on an on-going basis.

#### Inclusion criteria

Individuals eligible to take part in the study must be adults (age ≥ 18 years) and classified as overweight (BMI ≥ 25 kg/m^2^). These are individuals that would benefit from weight loss and for whom linear growth has stopped; therefore weight loss could not compromise growth. The study will include only people with learning disabilities in the mild to moderate range (assessed by the scoring system for “Ability & Development” scale [25]) and who live in the community. Unhealthy weight gain is more common in this specific population group than in individuals with severe to profound learning disabilities or individuals living in very restrictive environments such as institutions [5]. In addition, for individuals who receive regular care, a paid or informal carer must be willing to participate in the intervention.

#### Exclusion criteria

Individuals fulfilling one or more of the following criteria will be excluded: (1) acute mental illness requiring hospitalisation; (2) severe learning disabilities (assessed using the “Ability & Development” scale [25]); (3) substance misuse; (4) a confirmed diagnosis of Prader-Willi syndrome. These individuals would require a more specialised or intensive type of weight loss intervention, different from the Shape Up-LD programme.

#### Consent

Interested participants will be invited to an assessment appointment with a researcher at which informed consent will be sought from service users and, where applicable, their carers. Service users with mild learning disabilities who do not require regular care or assistance with shopping or preparing meals will be able to attend alone. Service users will have received an information sheet in the post beforehand and a full explanation will be given in person, with the opportunity to ask questions. The information sheets and consent forms have been developed in an accessible (i.e. easy-to-read) format with input from service users within the “Camden easy info group” of The Advocacy Project. Signed participant consent will be obtained face-to-face from both service users and carers taking part. The right of the participant to refuse to participate without giving reasons must be respected. All participants are free to withdraw at any time from the study without giving reasons and without prejudicing further treatment.

### Randomisation

Randomisation will be conducted by telephoning an external research coordinator at the Health Behaviour Research Centre, after the service user has provided informed consent and baseline data. RO (statistician) will generate the randomisation list. The technique of blocking will be employed to ensure equal numbers in the intervention and control arms. When enough dyads have been randomised to the intervention, a group will start the Shape Up-LD sessions.

### The interventions

#### Shape up- LD

The Shape Up-LD programme is a tailored version of Shape Up, a manualised healthy lifestyle programme that helps individuals learn new behaviours to manage their weight. This population-specific version of Shape Up was developed through a partnership between specialist dietitians of the Anglian Community Enterprise (ACE CIC) and service users with learning disabilities in North East Essex, a specialist dietician from Weight Concern and other learning disabilities professionals within the Camden NHS Trust and Learning Disability Partnership.

The original Shape Up programme involves participants attending one 60-min session every week for 8 weeks; however based on our field testing and prior experience of interventions in learning disabilities, we have extended the programme to run for 12 weeks. The additional four introductory sessions will give facilitators time to establish a rapport with the participants and provide a foundation for the remainder of the course, through focussing on basic healthy eating messages without any behavioural strategies. The remainder of the course will teach strategies for improving healthy eating and physical activity in line with the original Shape Up.

Shape Up is based on two theories: (1) “social cognitive theory” [26] and (2) “control theory” [27]. The intervention focuses on the concepts of self-control, self-efficacy and emotional coping responses. Behavioural techniques introduced in week 5 (and practiced throughout the subsequent weeks of the intervention) will include self-monitoring with the use of food and physical activity diaries, relapse prevention, assertiveness and goal setting.

Tailoring of Shape Up will include pictorial illustration of each session’s messages and use of simple spoken and written communication based on the recommendations of the Royal College of Nursing [28]. The photos used to develop the materials for each session and facilitate communication are part of the photo libraries Photosymbols [29] and the Greater Glasgow and Clyde LD Service [30].

The programme structure is as follows:

Introductory sessions:

Week 1: Getting to know each other and having fun with “food games”

Week 2: Food groups

Week 3: Food portions

Week 4: Thirsty for water (introduction to fluids)

Starting to Shape Up:

Week 5: Preparing to Shape Up: How to lose weight (plus goal setting and self-monitoring)

Week 6: Food groups (repeating messages from week 2 with more detail)

Week 7: Food portions and how to serve your food (repeating messages from week 3)

Week 8: Be Active (introduction to physical activity)

Week 9: Eating 3 meals and healthy snacks

Week 10: Feeling hungry (cravings and hunger) (relapse prevention, assertiveness)

Week 11: Shopping and cooking

Week 12: Things we have learned and keeping the weight off (weight maintenance)

While each session originally ran for 60 min, field testing suggested that this was not long enough. We have therefore extended the length of the sessions to 90 min. Where applicable, carers will also attend. Each session will be run at day centres once per week for groups of 4–6 service users and/or pairs of service users and their carers. The sessions will be led by two health professionals who have volunteered to be trained in the delivery of the intervention, at least one of whom will have clinical experience with people with learning disabilities.

Our experience of field testing the intervention and evidence from other studies [8] suggest that carers play an important role in weight management for adults with learning disabilities, and training carers could help them to engage with the programme and understand how best to support their service user. Therefore, carers who will be attending with the participant with learning disabilities will be given a half-day information/training session to introduce Shape Up-LD, address questions and explore how behaviour change can be supported. This training will be provided by the group facilitators prior to the first group session.

#### Control group

Usual care differs between services. Discussions with colleagues working in this area suggested that a brief one-off discussion about healthy eating and activity will approximately reflect what most individuals with learning disabilities currently receive. Therefore, each participant randomised to the control group will be invited to attend a session that will last 30–45 min. Where applicable, carers will be present at the session. A six-page booklet specially developed for people with learning disabilities that is commonly used by health professionals to provide health behaviour advice to service users will be used. The resource is accompanied by “facilitator notes” to assist health professionals in guiding service users through the booklet. The sessions will be delivered by the research assistant of the study.

The booklet covers the following topics: (1) the risks of putting on weight; (2) how to follow a healthy balanced diet based on the recommendations of the “Eat Well Plate” [31]; (3) the importance of regular eating patterns; (4) exercise advice based on the recommendations of current clinical guidelines [11]. Each participant will be given the specially designed booklets and a DVD (the Camden Audio-visual News DVD) that includes information on healthy eating and exercise [32]. The DVD has been developed for use by health professionals on healthy lifestyles and was produced in partnership with people with learning disabilities that live in Camden.

#### Training for staff delivering the interventions

A dietitian specialised in learning disabilities and trained by Weight Concern will train the professionals who will deliver the Shape Up programme and the usual care intervention. The training for Shape Up-LD will take place over 1 day followed by a half day observing the programme being delivered by the learning disability dietitians in a community setting in North East Essex. The facilitators who will deliver the usual care will be offered an hour training following the “facilitator notes” that explain how to use the booklet that will be used as part of the usual care intervention.

### Measures

#### Demographics

At baseline, demographic information including age, gender and ethnicity, and the Ability and Development score [25] will be recorded, as will medication use and level of support required.

#### Physical and biological

Participants will be asked to remove any belts, shoes, socks and heavy outer clothing for all measurements. Weight (kg) (measured to the nearest 100 g) and body fat (%) will be measured using TANITA TBF-300 MA Body Composition Analyser. Participants can opt to receive a printout of the results together with an explanation by the research team or not. Height will be measured, rounded up to the nearest centimetre, using the Leicester Height Measure, a standardised instrument for determining height. Waist circumference (cm) measurements will be based on the measurement protocol as recommended by the World Health Organisation (WHO) (2008) [33]. The waist will be measured at the midpoint between the top of the iliac crest and the lower margin of the last palpable rib in the mid auxiliary line. The measurement will be made over light clothing. Blood pressure (mmHg) will be measured using an automated BP monitor (Omron M2 Basic Upper Arm Blood Pressure Monitor) with the patient seated comfortably for 5 min prior to measurement and the arm supported at the level of the heart. All measurements will be taken twice. The final value will be calculated as the mean of the two measurements.

#### Psychological and behavioural

Service users will complete the following measures:

1. Mental health will be assessed using the “Psychiatric Assessment Schedule for Adults with a Developmental Disability” checklist (PAS-ADD) [34], a scale that has been used widely in this population and that has good psychometric properties.

2. Quality of life will be measured by QOL for the person with learning disabilities [35], which has been developed specifically for use in learning disabilities populations and has good internal consistency. It contains 40 items and has four subscales: ‘satisfaction’, competence/productivity’, ‘empowerment/independence’ and ‘social belonging/community integration’. We will measure a generic health-related quality of life using the EuroQol-5D (EQ-5D) [36], a five-item, three-level questionnaire, where the resulting 243 health states have associated preference scores obtained from the general population [37]. The EQ-5D is the generic health-related quality of life measure that is prescribed by NICE [38] for use in economic evaluations for the calculation of Quality-Adjusted Life Years (QALYs). The measure though has not been validated in patients with learning disabilities. The ED-5D will be validated against the QOL for the person with learning disabilities to inform our decision about how to measure quality of life in a main trial.

3. Self-esteem will be measured using the Rosenberg Self-Esteem Scale adapted for people with learning disabilities [39]. The questionnaire includes six statements followed by five answers rated as “never true” to “always true”. Scoring of the answers will identify participants with “positive self-esteem” and participants with “negative self-esteem”.

4. Knowledge and behaviour change for service users and their carers will be assessed using a simple checklist of the target messages and behaviours. The accessibility of the checklist was reviewed by service users from the Advocacy Project and revised based on their comments. We will also ask participants to bring in shopping receipts for the week prior to each assessment to measure changes in shopping behaviours and changes in costs of shopping.

5. Attitudes to healthy behaviours for service users and their carers will be assessed using an adapted measure from the Change4Life Survey [40]. The Change4Life Survey aims to detect changes in attitudes and behaviours attributed to the obesity prevention campaign programme Change4Life. The adapted questionnaire will focus on participants’ attitudes towards the lifestyle changes targeted by the Shape Up-LD programme.

6. The Client Service Receipt Inventory, learning disabilities version (CSRI-learning disabilities [41], will be used to measure costs. The first section of the questionnaire collects demographic information including place of residence and number of people sharing an accommodation and the second section collects information on medical conditions and use of medication. The rest of the questionnaire collects information on care package costs, use of direct payments by the service user, employment and benefits status. In addition, the questionnaire includes questions on daytime activities, e.g. voluntary work and adult education, use of hospital-based services and use of community-based services from the preceding 6 months.

#### Feasibility

We will record weekly recruitment rates, loss to follow-up, compliance rates (number of sessions attended) and basic costs. We will also record completion rates, number of incorrect responses, time taken to complete and the number of times a participant asks for help for each measure to assess acceptability and appropriateness.

#### Adherence to treatment protocol

Data on service provider adherence to the Shape Up-LD manual will be assessed by audiotapes of the sessions. All sessions will be audiotaped and transcribed for analysis and rated using a provider checklist based on the components of the intervention. These will be coded by an independent observer and a sub-group will be second coded to ensure reliability.

### Follow-up

Follow-up assessments of all physical/biological and psychological/behavioural measures will be done at appointments at the end of the treatment period (12 weeks) and then at 6 months to assess maintenance. The measurements will be taken by a trained researcher blind to group allocation.

#### Qualitative interviews

At the 12-week follow-up (end of the active treatment period), ten service users and/or service-user-carer dyads who received Shape Up-LD will be invited to take part in qualitative interviews. The interviews will be semi-structured and will follow an interview schedule that includes open-ended questions. The interviews will explore service user and carer experiences of Shape Up-LD including the acceptability of all elements of the trial, acceptability of the intervention itself, and barriers and/or facilitators to compliance with the intervention. Interviews will also be conducted with the service providers at 12 weeks to explore their experience of delivering the intervention.

### Statistics and data analysis

#### Sample size and assumptions

No RCTs have been carried out in this area, previous studies have used very different interventions and been of varying quality [6,23], and our own field testing of Shape Up-LD was on a very small sample. We are therefore unable to estimate the likely effect size of this intervention. In this feasibility trial, we will therefore recruit 60 participants. Our objective is to examine whether there is a clinically important change in the outcome due to the intervention. A 5% change in body weight is considered to be a clinically significant weight loss associated with health benefits [11].

#### Data analysis

As this is a feasibility trial, our main aim is to record the feasibility outcomes. We will also compare drop out rates in the experimental and comparator groups to assess the acceptability of randomisation. Acceptability of outcomes will be explored through the distribution of missing data among the measures. We will conduct exploratory statistical analyses to estimate effect sizes for a definitive trial, controlling for baseline characteristics such as baseline diet and activity levels, medication use and level of support. We will also calculate figures required for the sample size calculation of the definitive trial, for example, mean, SD and ICCs measuring clustering within groups.

#### Economic analysis

The EQ-5D will be validated against the QOL measure for the person with learning disabilities to give us an indication of the suitability of using the EQ-5D for the health economics analysis in a main trial. If the EQ-5D is found to be a suitable measure of QOL in learning disabilities patients, we will calculate the quality-adjusted life years for each patient using the standard formula from Dolan [37]. If not, we will investigate other strategies such as mapping or calculating cost per change in another quality of life measure.

We will calculate the costs associated with implementing and delivering Shape Up-LD. General resource use for the Shape Up-LD and usual care group, including carer time, will be collected using the CSRI and costed at national rates using the Personal Social Services Research Unit (PSSRU), Office for National Statistics (ONS) and reference cost data.

The planned cost effectiveness analysis is to examine the incremental cost per quality-adjusted life year gained (QALY) (ICER) of Shape Up-LD compared to usual care for the duration of the trial from the health-care and personal social services (PSS) perspective. Due to the small sample size there is likely to be a lot of uncertainty associated with the results and the trial will not be powered to detect differences between the two groups. We will use non-parametric methods though for calculating confidence intervals around the ICER based on bootstrapped estimates of the mean cost and QALY differences [42]. The bootstrap replications will also be used to construct a cost-effectiveness acceptability curve, which will show the probability that Shape Up-LD is cost-effective compared to usual care for different values of the NHS’ willingness to pay for an additional QALY gained. We will also subject the results to deterministic (one-, two- and multi-way) sensitivity analysis. A cost per unit change in weight will also be calculated and subjected to probabilistic and deterministic tests.

#### Qualitative analysis

All interviews will be audio recorded, anonymised and transcribed verbatim. The qualitative data software analysis package NVivo will be used to organise the transcripts and analyse the data. A thematic analysis will be used to identify the main themes that will form the basis of our results. The procedure of coding and development of a thematic map will be based on the recommendations of Braun and Clarke [43].

### Refining of materials

Both the qualitative and quantitative findings will be used to revise the Shape Up-LD materials, depending on the effectiveness of the programme. Findings will be fed back to the Trial Steering Committee, and their comments will guide the final draft to be used in a subsequent full-scale RCT.

### Ethical approval, research governance and trial sponsorship

Ethical approval was obtained from the Cornwall & Plymouth Research Ethics Committee via the Integrated Research Approval System (IRAS) (Ref. No. 12/SW/0089, Approval granted 05/04/12). Advice and support on governance and good clinical practice (GCP) issues will be provided by the PRIMENT Clinical Trials Unit, who will provide Standard Operating Procedures. The trial has been submitted to The International Standard Randomised Controlled Trials (ISRCT) and allocated the number ISRCTN 39605930. Camden & Islington NHS Foundation Trust is the sponsor of this trial.

## Discussion

There is currently a lack of weight management services tailored to the needs of learning disabilities service users. However, NICE Obesity Guidance [11] emphasises that advice, treatment and care relating to weight management should be accessible to people with learning disabilities. Provision of a population specific service would help to ensure more equitable health care for this group. Shape Up-LD has the potential to fill the current gap in weight management services for people with learning disabilities and could become widely used by NHS dietitians working within learning disabilities services. The manualised format of Shape Up-LD permits its replication in different settings, allows the training and supervision of health professionals following the strategies and techniques recommended in the manual, and facilitates an audit of the programme.

It is hoped that the proposed research will also benefit the health of NHS users, for example weight management may reduce the risk of cardiovascular disease and type 2 diabetes and improve mobility. Other predicted benefits to service users include improvements in general self-management abilities, quality of life and self-esteem. Carers may also benefit through increased knowledge of healthy behaviours, which could influence their own health behaviours and in turn impact the day-to-day care they provide, for example through changing shopping habits. The proactive, problem-solving approach used may also be empowering within the user-carer relationship [44]. Costs to the NHS may be reduced through decreases in use of services (e.g. GP visits, due to improved health of both learning disabilities service users and their carers). This study will lead to us refining the Shape Up- LD manual and materials, and we would hope that use of these resources would become an established part of local service delivery. A subsequent definitive trial could lead to more equitable weight management services for learning disabilities service users throughout the UK. This research will give us a better understanding of the feasibility of such a trial.

## Trial status

Recruitment will commence in September 2012 and is scheduled to continue for 12 months.

## Abbreviations

NHS: National Health Service; NICE: National Institute for Health and Clinical Excellence; BMI: Body mass index; MRC: Medical Research Council; RCT: Randomised controlled trial; CI: Confidence intervals; WHO: World Health Organisation; PAS-ADD: Psychiatric Assessment Schedule for Adults with a Developmental Disability” checklist; CSRI: Client Service Receipt Inventory; PSSRU: Personal Social Services Research Unit; ONS: Office for National Statistics; QALY: Quality-adjusted life year; IRAS: Integrated Research Approval System; GCP: Governance and good clinical practice; ISRCT: International Standard Randomised Controlled Trials

## Competing interests

The authors declare that they have no competing interests.

## Authors’ contributions

RJB, HC and SF conceived of the study and wrote the funding application with JW, MK, RO and AH. DS and RH have contributed to subsequent refinements of the design and economic analysis plan. RJB drafted the final protocol. DS is the trial manager and also contributed to the adaptation of the weight management intervention and materials. RO is the trial statistician. RH is the health economist for the trial. All authors revised the manuscript, and read and approved the final version.
